# A novel hyperthermophilic methylglyoxal synthase: molecular dynamic analysis on the regional fluctuations

**DOI:** 10.1038/s41598-021-82078-7

**Published:** 2021-01-28

**Authors:** Gyo-Yeon Seo, Hoe-Suk Lee, Hyeonsoo Kim, Sukhyeong Cho, Jeong-Geol Na, Young Joo Yeon, Jinwon Lee

**Affiliations:** 1 Department of Chemical and Biomolecular Engineering, Sogang University, Seoul, 04107 Republic of Korea; 2 C1 Gas Refinery R&D Center, Sogang University, Seoul, 04107 Republic of Korea; 3 Department of Biochemical Engineering, Gangneung-Wonju National University, Gangneung-si, Gangwon-do, 25457 Republic of Korea

**Keywords:** Industrial microbiology, Protein design, Enzymes, Protein design

## Abstract

Two putative methylglyoxal synthases, which catalyze the conversion of dihydroxyacetone phosphate to methylglyoxal, from *Oceanithermus profundus* DSM 14,977 and *Clostridium difficile* 630 have been characterized for activity and thermal stability. The enzyme from *O. profundus* was found to be hyperthermophilic, with the optimum activity at 80 °C and the residual activity up to 59% after incubation of 15 min at 95 °C, whereas the enzyme from *C. difficile* was mesophilic with the optimum activity at 40 °C and the residual activity less than 50% after the incubation at 55 °C or higher temperatures for 15 min. The structural analysis of the enzymes with molecular dynamics simulation indicated that the hyperthermophilic methylglyoxal synthase has a rigid protein structure with a lower overall root-mean-square-deviation value compared with the mesophilic or thermophilic counterparts. In addition, the simulation results identified distinct regions with high fluctuations throughout those of the mesophilic or thermophilic counterparts via root-mean-square-fluctuation analysis. Specific molecular interactions focusing on the hydrogen bonds and salt bridges in the distinct regions were analyzed in terms of interatomic distances and positions of the individual residues with respect to the secondary structures of the enzyme. Key interactions including specific salt bridges and hydrogen bonds between a rigid beta-sheet core and surrounding alpha helices were found to contribute to the stabilisation of the hyperthermophilic enzyme by reducing the regional fluctuations in the protein structure. The structural information and analysis approach in this study can be further exploited for the engineering and industrial application of the enzyme.

## Introduction

Methylglyoxal synthase (MGS, EC number 4.2.3.3) is a lyase for an efficient removal of a phosphate moiety from dihydroxyacetone phosphate (DHAP) to form pyruvaldehyde, an enol precursor that can be tautomerized spontaneously to methylglyoxal (MG)^1–4^. DHAP and MG are intermediates in a bypass of the cellular glycolytic pathway, where MG is converted to lactate and subsequently oxidized to pyruvate. However, in vivo millimolar concentration of MG is mutagenic and interferes with de novo protein and nucleic acid synthesis^3,5^; hence the function of MGS is emphasized to balance the physiological concentrations of the cytotoxic product. MGS has been found and characterized from gram-negative and gram-positive bacteria such as *Escherichia coli*^6^, *Pseudomonas saccharophila*^7^, *Proteus vulgaris*^8^, *Clostridium acetobutylicum*^9^ and *Bacillus subtilis*^10^, as well as *Saccharomyces cerevisiae*^11^ and thermophilic *Thermus sp*. GH5^5^.

MGS can be a valuable tool in the biochemical production of value-added chemicals from glycolytic intermediates, especially the commercial 1,2-propanediol^12–14^. 1,2-propanediol has applications as antifreeze, a feedstock for polyester resins in film and fiber manufacture, a substrate for optically active propylene oxide and polymers, and pharmaceutical products. In previous studies, MGS-expressing *E. coli* was metabolically engineered to produce MG, which was then converted to 1,2-propanediol^12,14^. Alternatively, enzymatic synthesis of MG from DHAP can be employed considering the cytotoxicity of the product. In this case, a highly stable and active MGS is sought for the industrial applications, where harsh conditions such as high temperature are often used. Advantages of the high temperature in industrial processes include improved mass transfer and reaction rates, increased substrate solubility, lower viscosity, and reduced risk of microbial contamination^15^. To this end, many industrial enzymes are isolated from thermophilic (optimum temperature at 50 °C ~ below 80 °C) or hyperthermophilic (optimum temperature at 80 °C or higher) organisms living in an extreme environment such as hot spring^16,17^. Furthermore, comparing the thermophilic or hyperthermophilic enzymes and the mesophilic counterparts in terms of amino acid sequences and three-dimensional structures can reveal the rationale for the increased thermostability because they share a high degree of structural similarity^16–18^. The rationale can be exploited to design tailor-made thermostable proteins^19,20^.

In this work, putative *mgsA* genes from *Oceanithermus profundus* DSM 14,977 and *Clostridium difficile* 630 encoding MGS enzymes were recombinantly expressed in *E. coli*, purified and characterized for optimum pH, optimum temperatures (T_opt_) and thermostability, where the MGS from *O. profundus* was found to be hyperthermophilic. The structural basis for the activity and thermostability of the enzymes has been analyzed via molecular modeling and discussed with respect to previously characterized MGSs.

## Results and discussion

### Screening, expression and purification for putative MGSs

Putative *mgsA* gene sequences were selected from the NCBI database by the sequence homology between 30 and 70% with the previously characterized MGSs such as those from *Escherichia coli* (ecMGS), *Bacillus subtilis* (bsMGS) and *Thermus sp*. GH5 (tsMGS). As a result, a MGS from *Clostridium difficile* 630 (cdMGS) with 45.3% similarity to ecMGS and a MGS from *Oceanithermus profundus* (opMGS) with 53.0% similarity to tsMGS were selected for characterization after initial screening of activity. The cdMGS consists of 137 amino acids with a molecular weight of 14.1 kDa, originating from the Gram-positive, anaerobic and spore-forming *Clostridium difficile* strain 630^21^. The opMGS consists of 125 amino acids with the molecular weight of 13.9 kDa, and its gene is from the thermophilic bacterium *Oceanithermus profundus* isolated from a deep-sea hydrothermal vent^22^.

The *mgsA* genes were cloned along with a Strep tag for affinity purification, which is known to have a minimal effect on the main protein body (See Supplementary Table S1 online)^23,24^. The result of protein expression, Strep-affinity purification and SDS-PAGE clearly showed purified polypeptides with appropriate sizes for cdMGS and opMGS (See Supplementary Fig. S1 online). Furthermore, a six-aspartate tag (Asp tag) was added to the N-terminal of opMGS to increase its solubility^25^.

### Specific activity of cdMGS and opMGS

**Figure 1:**
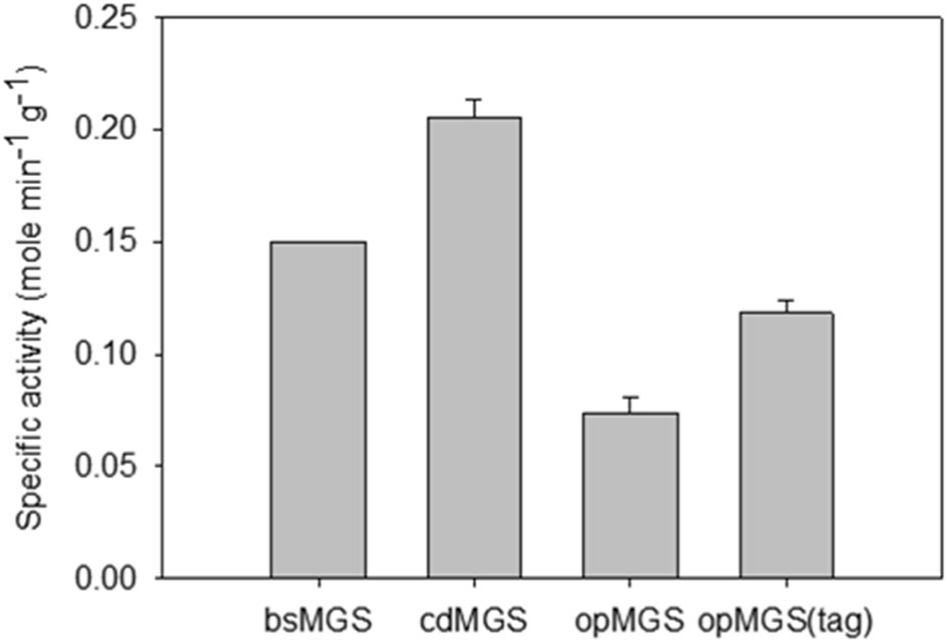
Specific activities of the purified MGSs at pH 7.5, 30 °C. Each value and error bar represents the mean and the standard deviation of at least three independent trials, respectively. The specific activity of bsMGS from *Bacillus subtilis* has been reported previously and presented here for comparison^4^.

Figure 1 shows the specific activities of the purified MGSs measured at 30 °C and pH 7.5, along with bsMGS, a mesophilic MGS used for 1,2-propanediol synthesis with the highest known specific activity described previously^4^. The specific activities of cdMGS and opMGS were 0.21 and 0.07 mol min^−1^ g^−1^, respectively. The cdMGS had 36.7% higher specific activity than that of bsMGS with 0.15 mol min^−1^ g^−1^. The activity of opMGS was not as high as those of bsMGS or cdMGS, but the addition of the Asp tag increased the activity to 0.12 mol min^-1^ g^-1^. Explaining the delicate effect of a tag on the enzymatic activity when the tag is distant from the active site remains a challenging task, although there are reports of net charge affecting the solubility and the activity of proteins^26,27^.

### Effect of pH on the activity of cdMGS and opMGS

**Figure 2:**
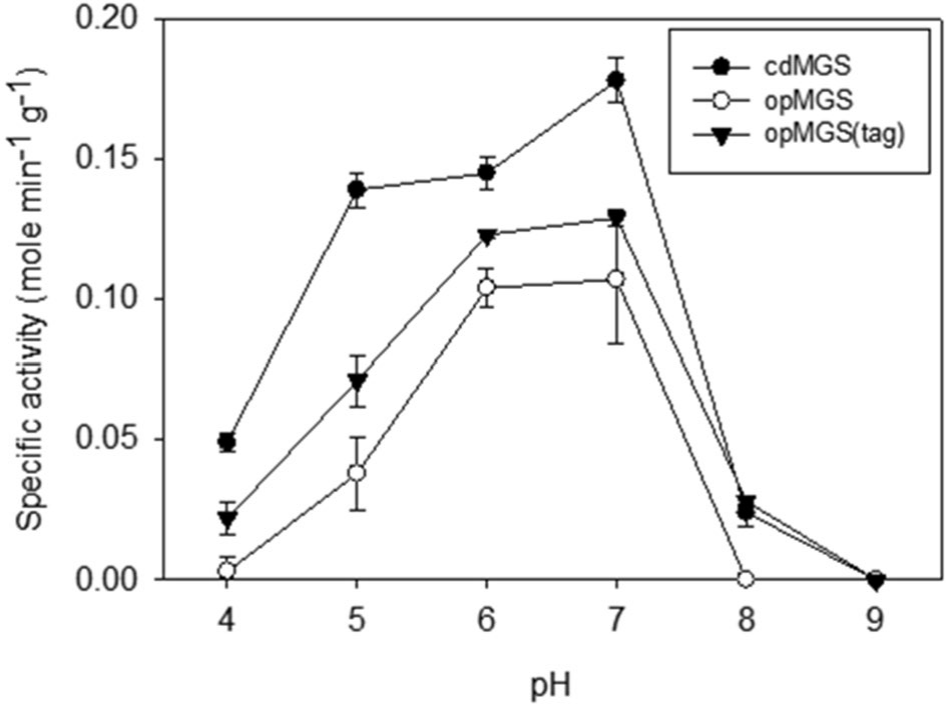
pH optima for the purified MGSs. Each value and error bar represents the mean and the standard deviation of at least three independent trials, respectively.

Optimal pH for the cdMGS, opMGS and opMGS (Asp tag) were investigated, which showed all three MGSs had the highest specific activity at pH 7 (Fig. 2). The cdMGS retained relatively high specific activity within the pH range of 5–7 but the activity decreased sharply outside the range. The optimal pH range was narrower for both opMGS and opMGS (Asp tag) than that for cdMGS. The three MGSs seemed to be deactivated below pH 4 or above pH 8. These are comparable to the previously reported, neutral pH optima for MGSs from *E. coli* (pH 7.5)^6^, *Thermus sp.* GH5 (pH 6)^5^, *Capra hircus* (pH 7.2)^28^, *Clostridium acetobutylicum* (pH 7.5)^9^, *Proteus vulgaris* (pH 7.7)^8^, but different from the alkalophilic MGSs from *Pelomonas saccharophilia* (pH 8.2)^7^ and *Saccharomyces cerevisiae* (pH 9.5–pH 10.5)^11^.

### Temperature optima and thermostability of cdMGS and opMGS

**Figure 3:**
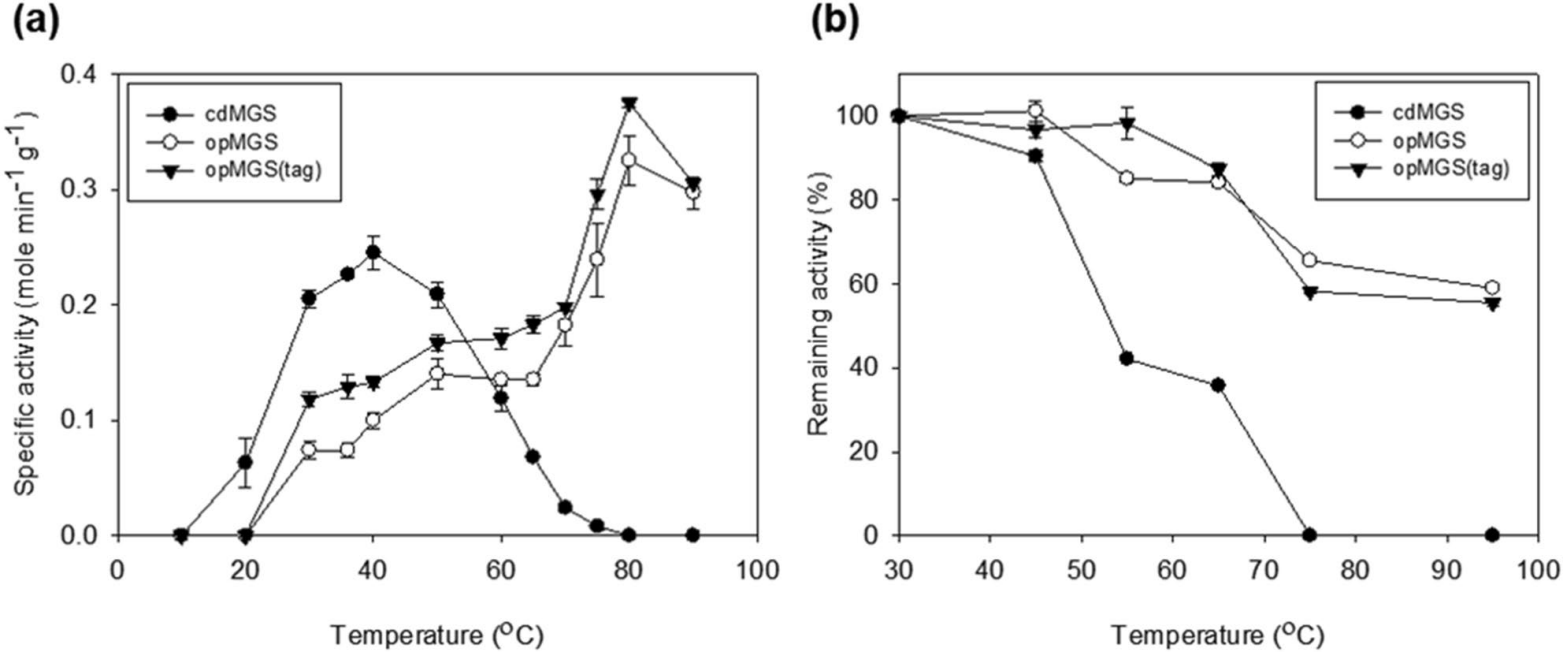
Thermal properties of the purified MGSs. (**a**) Temperature optima; (**b**) Thermostability. Each value and error bar represents the mean and the standard deviation of at least three independent trials, respectively.

The T_opt_ for cdMGS and opMGS were determined by carrying out the enzymatic reaction with the substrate incubated under various temperatures (Fig. 3a). The cdMGS showed the highest activity at 40 °C, while opMGS and opMGS (Asp tag) had T_opt_ at 80 °C. The thermostability of the enzymes was also determined by incubating the enzymes at different temperatures before the activity assay (Fig. 3b). The cdMGS maintained 90% remaining activity up to 45 °C but the activity rapidly decreaseed to less than half at 55 °C or higher temperature. In contrast, opMGS and opMGS (Asp tag) had much higher thermal stabilities. They retained over 85% and 98% activities at 55 °C, and 59% and 55% activities at 95 °C, respectively. The results indicate opMGS has a higher thermostability than the previously studied tsMGS from *Thermus sp.* GH5, which had T_opt_ at 75 °C and a rapidly decreasing activity over 75 °C^5^. The tsMGS presented approximately 10% remaining activity at 95 °C. By definition, tsMGS is a moderate thermophilic enzyme, while opMGS is a hyperthermophilic enzyme with T_opt_ at 80 °C or higher^16^.

### Sequence comparison of MGSs

**Figure 4:**
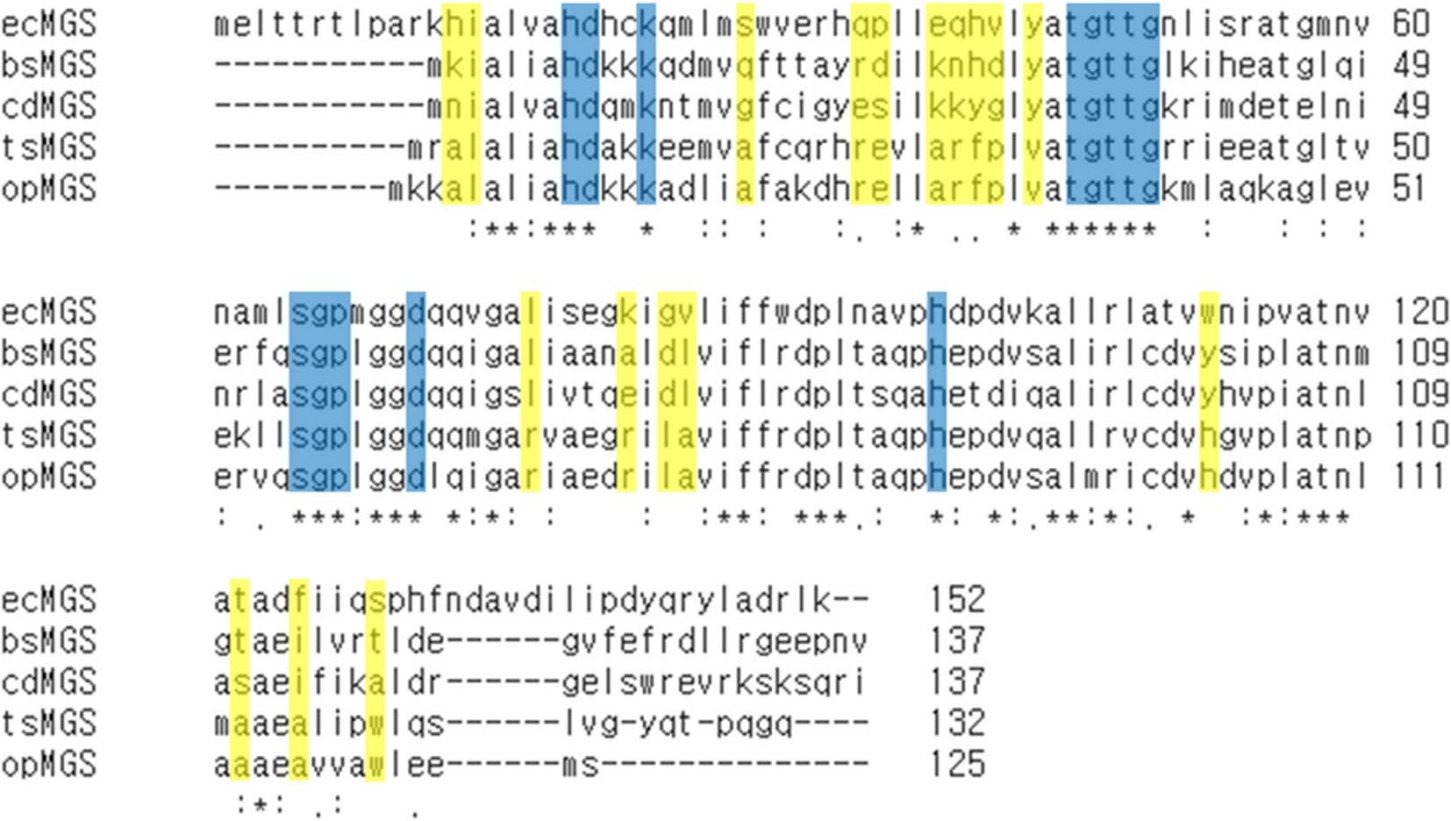
Amino acid sequence alignment of cdMGS and opMGS with ecMGS (*E. coli*), bsMGS (*B. subtilis*) and tsMGS (*Thermus sp*. GH5). The ecMGS, bsMGS and cdMGS represent mesophilic MGSs, and tsMGS and opMGS represent thermophilic MGSs. Blue-shades indicate the active site. Yellow-shades indicate residues that are found common in the thermophilic MGSs but different from the mesophilic enzymes. Asterisk, colon and full stop indicate the degree of sequential similarity from the highest to the lowest.

Alignment of the amino acid sequences from the mesophilic MGSs (ecMGS, bsMGS and cdMGS) and the thermophilic MGSs (tsMGS and opMGS) indicates the catalytic function of specific residues and sequential differences responsible for the difference in the thermostability (Fig. 4). The homology between cdMGS and opMGS was 49%. The thermophiles, especially the hyperthermophilic opMGS, are shorter toward the C-terminal than the mesophilic enzymes, generating more compact structures. The active site residues identified from ecMGS and bsMGS are highly conserved among all five MGSs^3,4^. D71 (numbering in ecMGS) is known to play a key catalytic role as a base to withdraw a *pro*-S proton from the C_3_ atom of DHAP, while H19 can stabilize the O_3_ hydroxyl group via hydrogen bonding. H98 helps the transfer of a proton to the O_2_ to form an enediol, although the function of H98 was suggested to be not critical in the catalytic mechanism itself but focused more on the regulation of the MGS conformation between active and inactive states^29–31^. K23, S65 and T45–G49 surround and stabilize the phosphate moiety binding via hydrogen bond and salt bridge, and G66–P67 follow the S65 in a loop covering the C_1_ and C_2_ atoms of DHAP. The residues that are present only in the thermophilic MGSs could be identified, many of which were hydrophobic residues in replacement of the hydrophilic homologues of the mesophiles. This may be used in thermostability engineering, although their specific molecular functions must be analyzed further.

### Docking of DHAP in the active sites of the MGSs

**Figure 5:**
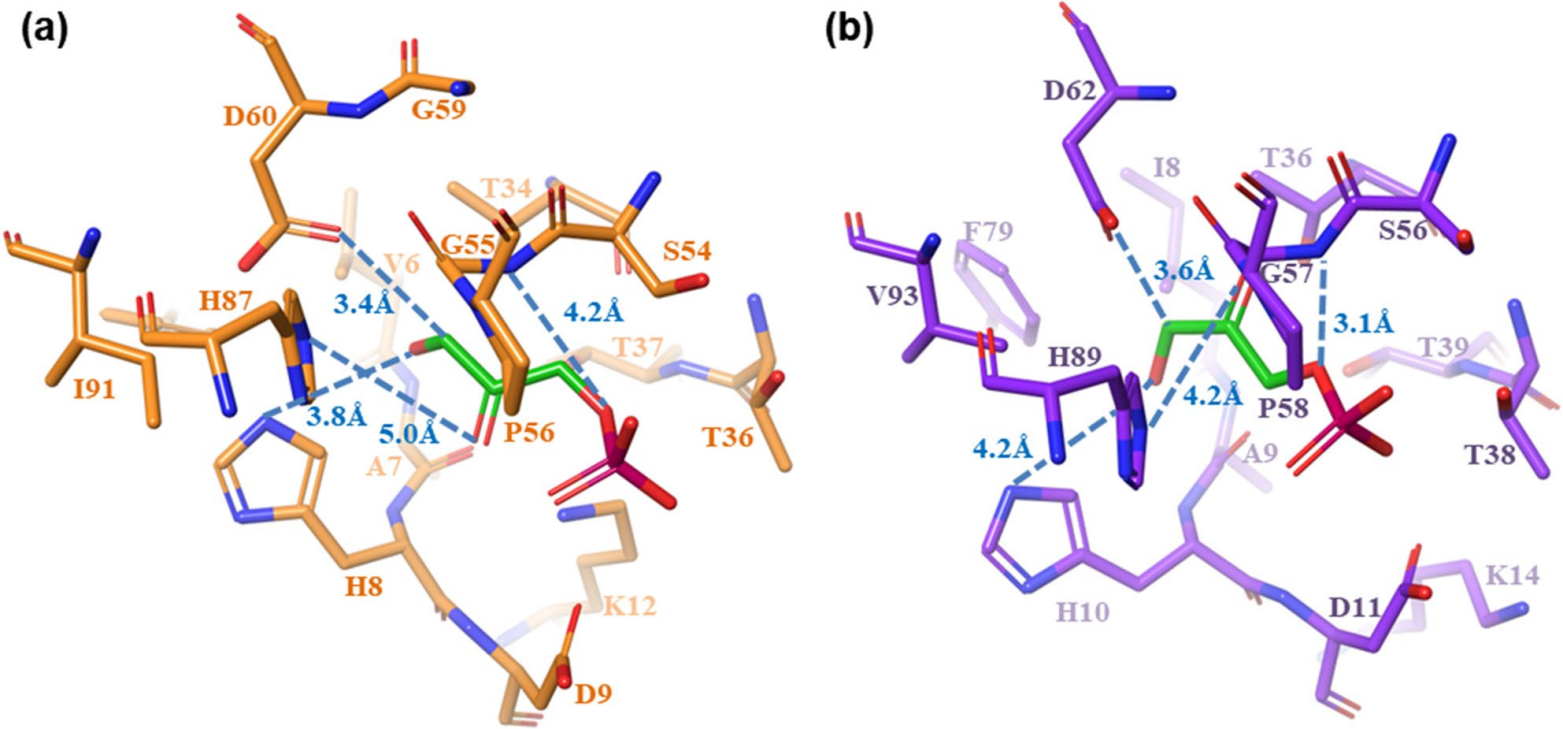
DHAP-docked enzymatic active sites. (**a**) cdMGS; (**b**) opMGS. The carbon atoms of DHAP, cdMGS and opMGS are colored as green, orange and purple, respectively. Nitrogen, oxygen, and phosphorous atoms are colored as blue, red and magenta, respectively. Distances between the key residues and substrate atoms are marked by dashed lines. ΔG_bind_ for DHAP in cdMGS and opMGS were − 3.76 and =  − 3.01 kcal mol^−1^, respectively.

The three dimensional structures of cdMGS and opMGS have been constructed by homology modeling. The active sites share a high similarity, yet the configuration of the substrate docked, DHAP, showed disaccord between the two MGSs (Fig. 5). The role of D71 in ecMGS as a catalytic base is played by D60 in cdMGS and D62 in opMGS^30^. The hydroxyl group at O_3_, oxidized to a ketone during the proton transfer, is stabilized via H8 (cdMGS) and H10 (opMGS). The H98 in ecMGS is equivalent to H87 and H89 in cdMGS and opMGS, respectively, to assist the stabilization of the O_2_ oxyanion and the transfer of a proton. The accessorial but not critical function is reflected in the relatively longer distance between the residue and the O_2_ atom than other critical catalytic distances. The backbone N from G55 (cdMGS) and G57 (opMGS) form a hydrogen bond with the phosphate oxygen where the C-O bond is lysed. The conserved motif of TGTTG, lysine and serine (residue no. 34-38, 12 and 54 for cdMGS, and no. 36-40, 14 and 56 for opMGS) are also used to stabilise other oxygens of the phosphate group via salt bridges and hydrogen bonds. The binding energies (ΔG_bind_) for the substrate were − 3.76 and − 3.01 kcal mol^−1^ for cdMGS and opMGS, respectively, which indicated a more stable binding in the active site of cdMGS for a lower *K*_*M*_.

### Structural basis for the thermostability

**Figure 6:**
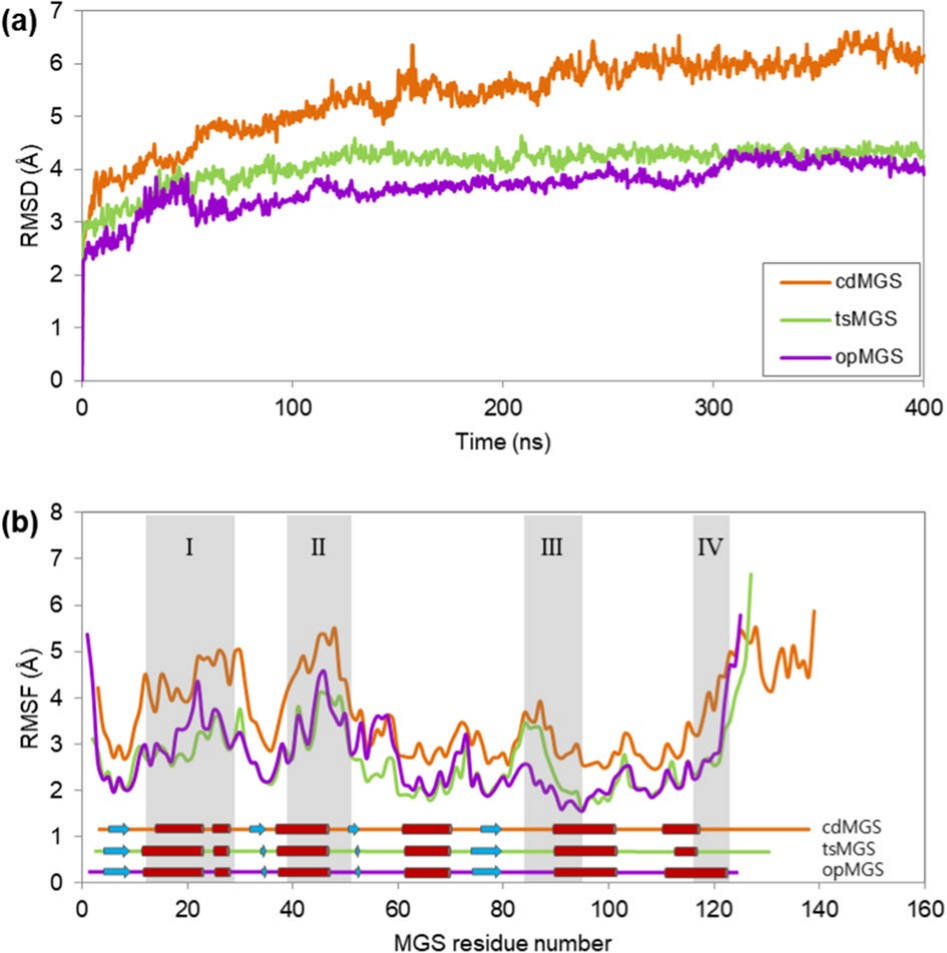
Molecular dynamics of opMGS, tsMGS and cdMGS. The MD simulation was carried out at 358 K for 400 ns. (**a**) Overall root-mean-square-deviation (RMSD) of the MGSs; (**b**) Root-mean-square-fluctuation (RMSF) for each residue of the MGSs. The three MGS sequences were aligned and a common set of residue numbers was assigned for the RMSF analysis of the homologous residues. Secondary structures such as α-helices (red cylinders), β-strands (blue arrows) and loops (lines with corresponding colors of the MGSs) are indicated. Regions of high difference in fluctuation between cdMGS and opMGS are shown as shaded boxes (I–IV).

In order to examine the molecular basis for the increased thermostability, the structures of hyperthermophilic opMGS, thermophilic tsMGS and mesophilic cdMGS have been subjected to molecular dynamics simulation (MD) of 400 ns at 353 K temperature. The overall molecular root-mean-square deviation (RMSD) was the lowest in opMGS, followed by tsMGS and cdMGS, indicating the structural rigidity was higher toward the more thermostable MGSs (Fig. 6a). In-depth residue-by-residue analysis of the structural rigidity was also carried out by calculating the root-mean-square fluctuation (RMSF) across the MGS residues (Fig. 6b). Four Regions (I–IV) of large difference in the structural rigidity among the MGSs were identified by calculating the RMSF difference between the thermophilic MGSs and cdMGS, and whether the residue RMSF difference was larger than the average RMSF difference. Each Region mainly encompasses alpha-helices, such that the Region I surrounds the alpha-helices α1–α2, the Region II for the α3 and the following loop, the Region III for the loop between β4 and α5 and a half of α5, and the Region IV for a part of α6 and the following loop toward the C-terminal. These helices surround the core beta-sheet from both sides, where the mostly hydrophobic beta-sheet shows a relatively similar rigidity in the thermophilic and mesophilic MGSs.

Several factors increasing the stability of proteins in thermophiles have been suggested previously, such as atomic packing, loop shortening and increased number of salt bridges^32–34^. Specific molecular interactions that could enhance the structural rigidity in the four Regions of hyperthermophilic opMGS focusing on salt bridges and hydrogen bonds were therefore analyzed. Disulfide bonding that is widely found in thermophilic archaean proteins was not found in the bacterial opMGS and tsMGS, with a limited number of cysteine residues positioned distant from each other^35^. It has been reported not all thermophilic organisms contain an abundance of disulfides, especially the methanogens and sulfur-reducing chemolithotrophs growing at strongly reducing conditions which necessitate the preclusion of cytosolic disulfide bonding^36^. However, utilization of the MGSs in industry can be free from such restriction, and may be engineered with disulfides to further increase the thermal stability.

**Figure 7:**
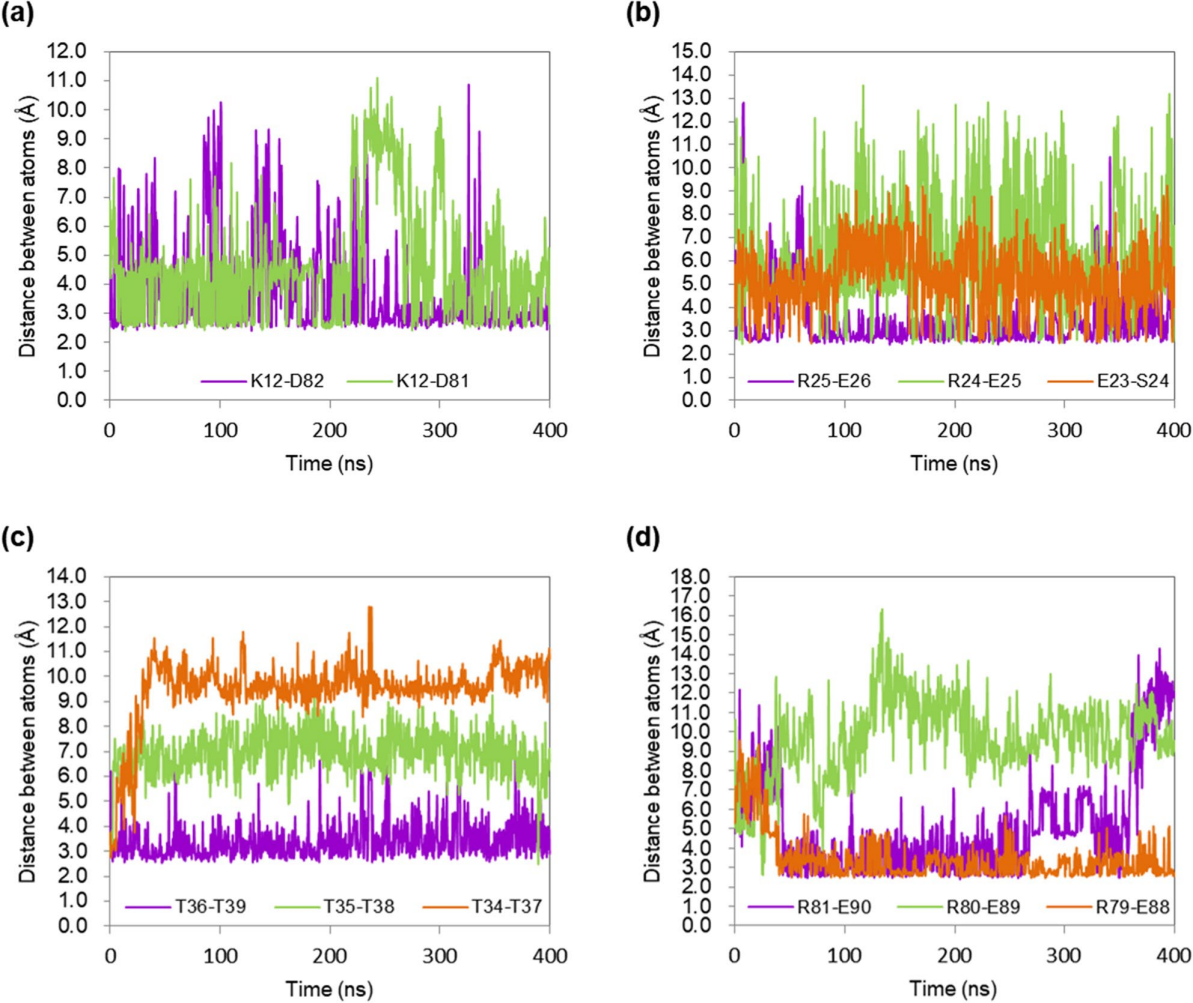
Interaction distance between residues that can affect RMSF in MGSs. Purple, green and orange correspond to the interactions between residues at homologous positions in opMGS, tsMGS, and cdMGS, respectively, with the residue number insets. (**a**) Salt bridges between specific lysines and aspartates in opMGS and tsMGS. The lysine is replaced by a methionine and cannot form a salt bridge in cdMGS; (**b**) Salt bridges between specific arginines and glutamates in opMGS and tsMGS. The salt bridge is replaced by a hydrogen bond between glutamate and serine in cdMGS; (**c**) Hydrogen bonds between specific threonines in the three MGSs; (**d**) Salt bridges between specific arginines and glutamates in the three MGSs.

**Figure 8:**
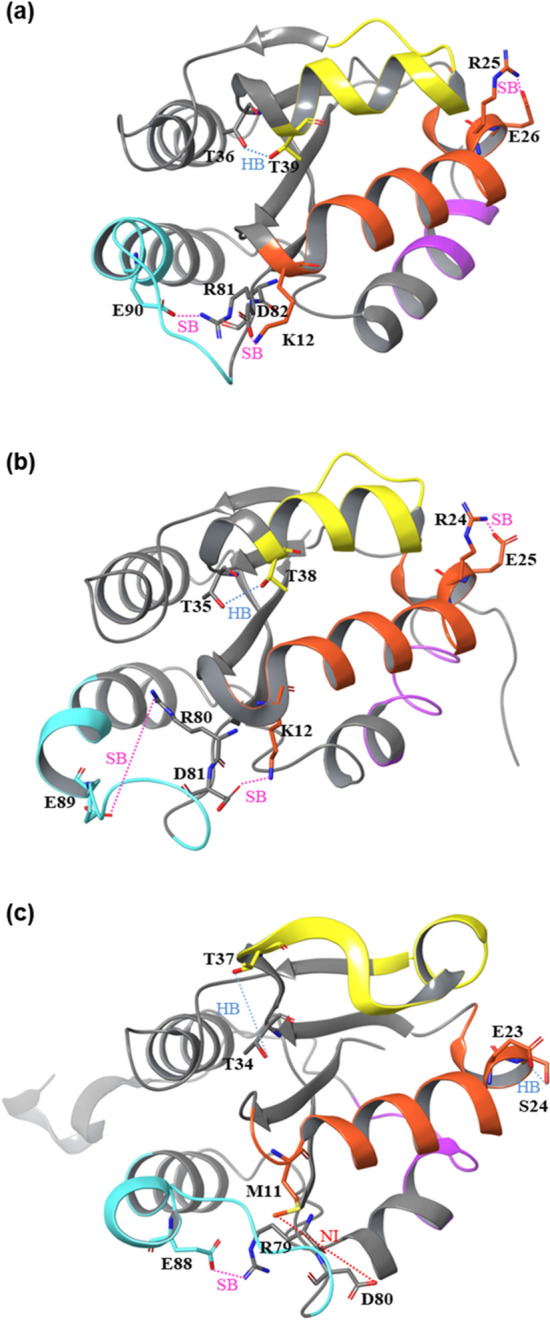
Modeled structures of the MGSs. (**a**) opMGS; (**b**) tsMGS; and (**c**) cdMGS. The regions of high RMSF difference are colored as orange (I), yellow (II), cyan (III) and purple (IV). The conformation of residues between which the distances are measured are indicated with the expected interactions such as salt bridge (SB), hydrogen bond (HB) and no corresponding interaction (NI).

Figures 7 and 8 show the distances between residues that could form the salt bridges or hydrogen bonds. K12-D82 in opMGS can form a salt bridge and maintain them at 353 K in a reasonable frequency to increase the interaction of the α1 helix in the Region I and the loop shortly after the β4, whereas a corresponding bridge cannot be formed by M11 and D80 or nearby residues in cdMGS (Fig. 7a). The aspartate is conserved among the five MGSs in the sequence alignment. A11 in tsMGS is at the homologous position to the K12, but the adjacent K12 side chain is within the bridge-forming distance from the aspartate. R25-E26 in opMGS or corresponding residues in tsMGS and cdMGS can form salt bridges that increase the stability of the short α2 helix in the Region I, but much higher frequency was observed in opMGS than in tsMGS or cdMGS (Fig. 7b). The two residues are adjacent and not involved in an inter-secondary structure interaction, but instability in the α2 can lead to increased fluctuations in the α1 because the α2 is right at the end of the α1 in a bent conformation, holding α1 in place. The hydrogen bond between T36-T39 can be observed at higher frequency in opMGS, whereas the distances were longer in tsMGS and cdMGS, hardly forming the bond (Fig. 7c). Although these residues were conserved in the sequence alignment of the five MGSs, the α3 distorts extensively in cdMGS, leading to the increased distance (Fig. 8c). The T36-T39 hydrogen bond can increase the interaction between the β2 and the α3 in the Region II, contributing to the local rigidity in opMGS. R81-E90 in opMGS and R79-E88 in cdMGS can form salt bridges between the loop following the β4 and the α5 in the Region III, but the effect on the rigidity of the region can be different in opMGS and cdMGS because R79 in cdMGS is located in a loop with higher flexibility due to lack of an interaction between M11 and D80 (Fig. 7d). Interestingly, tsMGS could not form a salt bridge at this location that may contribute to the lowered stability than that of opMGS. In combination with the K12-D82 bridge found in both opMGS and tsMGS but not in cdMGS, it is implicated that the K12-D82 bridge is important for being a thermophile, and the R81-E90 bridge is important for being a hyperthermophile in the MGSs. Other hydrogen bonds and salt bridges have been extensively searched throughout the MGS structures and their interatomic distances measured, but no significant difference between the hyperthermophilic, thermophilic and mesophilic MGSs were observed. This includes the Region IV, and its high fluctuation in cdMGS compared to the thermophiles could be derived from its extended C-terminal loop rather than from the absence of specific molecular interactions.

Overall, the increased thermal stability in tsMGS and opMGS could be explained by a rigid beta-sheet core and capability of alpha-helices to interact with the core via salt bridges and hydrogen bonds that specifically reduce regional fluctuations.

## Conclusion

In this study, a hyperthermophilic MGS has been discovered and structurally analyzed for its high thermostability. Two bacterial methylglyoxal synthases from *C. difficile* 630 and *O. profundus* were cloned, expressed and characterized, of which cdMGS was mesophilic with T_opt_ at 40 °C and opMGS was hyperthermophilic with T_opt_ at 80 °C. The opMGS presented the highest known T_opt_ and thermostability among MGSs characterized so far. These newly characterized MGSs can be added to the set of enzymes for utilization in industry to produce value-added chemicals. Furthermore, substrate docking and molecular dynamics simulation were employed to explain their activity and stability. Identification of key interactions including specific salt bridges and hydrogen bonds that restrict the regional fluctuations in MGSs can be exploited to further engineer the enzymes for industrial purposes.

## Methods

### Gene cloning

The *mgsA* genes from *Clostridioides difficile* 630 (Genbank ID: AJP10844.1) and *Oceanithermus profundus* DSM 14,977 (Genbank ID: ADR35584.1) encoding the MGS enzymes were synthesized by Bioneer Co. (Republic of Korea). For affinity chromatography-mediated protein purification, a Strep tag consisting of a nine residue-long polypeptide was cloned via PCR to the N- or C-terminal of the MGSs with specific primers (Supplementary Table S1). A six-aspartate tag was also added to the N-terminal of opMGS to increase the solubility of the protein. PrimSTAR Max DNA polymerase (Takara, Japan) was used for the PCR following the manufacturer’s protocol. The pETduet-1 vector was linearized with QuickCut NdeI & KpnI restriction enzymes (Takara, Japan) before ligation with the PCR products using an In-Fusion HD Cloning Kit (Clontech, USA). The constructed vectors were transformed into competent *E. coli* DH5α (RBC, Taiwan) and selected on LB agar plates supplemented with 100 µg ml^−1^ ampicillin as a marker. The cells were grown in 50 ml LB medium supplemented with 100 μg ml^−1^ ampicillin at 37 °C, 200 rpm in a shaking incubator. The vectors were mini-prepped and their sequences were confirmed by DNA sequencing.

### Protein expression and purification

The cloned expression vector-transformed *E. coli* BL21 were selected on LB agar plates and cultivated in 50 ml LB medium at 37 °C, 200 rpm, each supplemented with 100 μg ml^−1^ ampicillin to an optical density_(600 nm)_ of 0.5–0.7. The cultures were then induced with 80 µM isopropyl-ß-thiogalactopyranoside (IPTG) at 20 °C, 200 rpm for 19 h. The cells were harvested by centrifugation at 4500×*g*, 4 °C for 10 min and stored at − 80 °C as cell pellets for further uses.

For total protein extraction, the cell pellets were re-suspended in sonication buffer (Biosesang, Korea) and incubated 20 min on ice. These solutions were sonicated on ice with Ultrasonic Generator (20 kHz, 700 W, ULSSO HI-TECH CO., Republic of Korea) setting 50% power, 2 s on/3 s off program until cell lysates were obtained. The cell lysates were centrifuged at 10,000×*g* for 15 min at 4 ℃ to discard cell debris. Clear supernatants were applied to affinity chromatography using Strep-Tactin resin (IBA, Germany) according to the manufacturer’s recommendations to purify the MGS enzymes^23,24^. Protein Assay Kit (Bio-Rad, USA) was used to quantify the purified proteins.

### SDS-PAGE analysis

The insoluble total protein, soluble total protein and purified MGSs from each strain were prepared as samples during the protein purification steps. All samples were diluted to a final concentration of 10 µg protein per 16 µl and mixed with 4 µl of 5 × SDS-PAGE sample buffer (Biosesang, Korea). After boiling (5 min, 95 °C), the samples were cooled on ice. 20 µl of the samples or 7 µl of ProSieve Color Protein Marker (Lonza, Swiss) were loaded to each lane of 4–12% Tris gels (Bio-Rad, USA). Gel electrophoresis was carried out in Tris–glycine-SDS buffer (0.3% Tris, 1.4% glycine, 0.1% SDS (w/v)) at 200 V using PowerPac Basic Power Supply (Bio-Rad, USA). Coomassie Brilliant Blue G-250 Staining Solution (3 M, USA) was used to stain the gels with microwaves, followed by destaining for 1–2 h in 30% methanol / 10% acetic acid (v/v) destaining buffer with shaking (50 rpm).

### MGS activity assay

MGS activity assay using spectrophotometric determination of MG was described previously^10,37^. In more detail, a reaction mixture containing 0.44 ml of 40 mM reaction buffer, 50 µl of 30 mM DHAP and 10 µl of 10 µg ml^-1^ purified MGS were incubated at 30 °C for 10 min in Thermomixer (Eppendorf, USA). After the reaction, 100 µl of each reacted solution was transferred to a mixture of 0.9 ml H_2_O and 0.33 ml 2,4-dinitrophenylhydrazine (0.1% (w/v) 2,4-dinitrophenylhydrazine in 2 N HCl) and were incubated at 30 °C for 15 min. Finally, 1.67 ml of (w/v) NaOH solution was added and additionally incubated at 30 °C for 15 min. The optical density at 555 nm was determined to measure the MG concentration using a molar extinction coefficient of 4.48 × 10^4^.

### Buffers for determining the effect of pH on the MGS activity

The purified enzymes were dialyzed and re-suspended in various buffers ranging from pH 4–9 to investigate the effect of pH on the MGS activities and identify the pH optima. 40 mM succinic acid-NaOH buffer (pH 4–6), imidazole–HCl buffer (pH 7), tris-(hydroxymethyl)-aminomenthane buffer (pH 8) and glycine–NaOH buffer (pH 9) were used as reaction buffers, from which an optimum buffer and pH was selected.

### Temperature optimum and thermostability of cdMGS and opMGS

Buffers were first prepared at the temperature of use to minimize pH change over the different temperatures. In order to determine the optimum temperatures (T_opt_) for the purified MGSs, the substrate in 40 mM imidazole–HCL buffer (pH 7) was pre-incubated at desired temperatures (10–90 °C) for 10 min before the enzyme was added and incubated for 5 min and the MGS activity assay was carried out. For the thermostability determination, each purified MGS was incubated at different temperatures (30–95 °C) for 15 min and then immediately cooled on ice^5^. The remaining activity was then measured at 30 °C, pH 7 as described in the MGS activity assay method.

### Molecular modeling

Hexameric enzyme structures of opMGS and tsMGS were homology-modeled based on the crystal structure of the MGS from *T. thermophiles* HB8 (PDB ID: 1WO8), while that of cdMGS was modeled on the MGS from *E. coli* (PDB ID: 1EGH) with the Maestro Prime module from Schröedinger (New York, NY, USA) as previously described^38,39^. DHAP structure was created with all possible ionization states via the LigPrep module and docked in the opMGS and cdMGS models via the standard precision mode of the Glide module in the Maestro software^40,41^. The opMGS, tsMGS and cdMGS structures were subjected to Desmond molecular dynamics simulation by creating TIP3P hydration models with OPLS2005 forcefield^42^. The production type was NVT, and the simulation consisted of 400 ns duration at 358 K temperature. The trajectories of the enzymes were saved every 400 ps, producing 1,000 frames for analysis.

## Supplementary Information

Supplementary Information.
