# Supplementary material for: A novel hyperthermophilic methylglyoxal synthase: molecular dynamic analysis on the regional fluctuations
Source: Sci Rep. 2021 Jan 28;11:2538. doi: 10.1038/s41598-021-82078-7 (PMC7843640; doi:10.1038/s41598-021-82078-7)
Supplement: Supplementary file 1 — Supplementary Information. [file 41598_2021_82078_MOESM1_ESM.docx]

**Supplementary Information**

A novel hyperthermophilic methylglyoxal synthase: molecular dynamic analysis on the regional fluctuations

Gyo-Yeon Seo^1,a^, Hoe-Suk Lee^3,a^, Hyeonsoo Kim^1^, Sukhyeong Cho^2^, Jeong-Geol Na^1^, Young Joo Yeon^3,*^, Jinwon Lee^1,2,*^

^1^Department of Chemical and Biomolecular Engineering, Sogang University, Seoul, 04107, Republic of Korea

^2^C1 Gas Refinery R&D Center, Sogang University, Seoul 04107, Republic of Korea.

^3^Department of Biochemical Engineering, Gangneung-Wonju National University, Gangneung-si, Gangwon-do, 25457, Republic of Korea

^a^These authors contributed equally to this work.

*Corresponding authors: Young Joo Yeon (TEL: +82-33-640-2401, FAX: +82-33-641-2410, Email: [yjyeon@gwnu.ac.kr](mailto:yjyeon@gwnu.ac.kr), Jinwon Lee (TEL: +82-2-705-8919, FAX: +82-2-702-7926, Email: [jinwonlee@sogang.ac.kr](mailto:jinwonlee@sogang.ac.kr)

**Supplementary Table S1.** Primers, vectors and strains used in this study.

| **Primer, vector and strain name** | **Property** | | |
| --- | --- | --- | --- |
| **Primer** | **Sequence (5’ to 3’ direction)**^a^ | **For cloning:** |  |
| CM_For | *ATAAGAAGGAGATATA***CATATG**ACAGTCCTGAAAAGGAGCATCACCGATGTGGAGCCACCCGCAGTTCGAAAAAATGAATATAGCATTAGTAGCACAT | pETCM01 |  |
| CM_Rev | *TTTCTTTACCAGACTCGAG***GGTACC**TTAAATACGTTGACTTTTGCTTTT | pETCM01 |  |
| OM1_For | *ATAAGAAGGAGATATA***CATATG**AGCCCCTTCGAAAGGAGCATCTACCATGTGGAGCCACCCGCAGTTCGAAAAAATGA AAAAGGCGCTGGCGCTG | pETOM01 |  |
| OM1_Rev | *TTTCTTTACCAGACTCGAG***GGTACC**TCAGCTCATCTCCTCGAGCCA | pETOM01 |  |
| OM2_For | *ATAAGAAGGAGATATA***CATATG**AGCCCCTTCGAAAGGAGCATCTACCATGAAAAAGGCGCTGGCGC TG | pETOM02 |  |
| OM2_Rev | *TTTCTTTACCAGACTCGAG***GGTACC**TCATTTTTCGAACTGCGGGTGGCTCCACATGCTCATCTCCTCGAGCCA | pETOM02 and pETOM03 |  |
| OM3_For | *ATAAGAAGGAGATATA***CATATG**AGCCCCTTCGAAAGGAGCATCTACCATGGACGATGATGACGACGATAAAAAGGCGCTGGCGCTG | pETOM03 |  |
| **Vector** | **Gene and tag sequence inserted** | | |
| pETduet-1 | No gene or tag inserted | | |
| pETCM01 | pETduet-1::N-terminal Strep-*mgsA* of *C. difficile* 630 | | |
| pETOM01 | pETduet-1::N-terminal Strep-*mgsA* of *O. profundus* | | |
| pETOM02 | pETduet-1::C-terminal Strep-*mgsA* of *O. profundus* | | |
| pETOM03 | pETduet-1::N-terminal Asp tag -C-terminal Strep-*mgsA* of *O. profundus* | | |
| **Strain** |  | | |
| *E. coli* DH5a | F^–^ *(80d lacZ M15) (lacZYA-rgF)U1691 hsdR17(m*^+^*) recA1 endA1 relA1 deoR* | | |
| *E. coli* BL21 | F^–^ *dcm ompT hsdS(rB* ^–^ *mB* ^–^ *) ga* | | |
| MGS00 | *E. coli* BL21 harboring pETdue**t**-1 (control) | | |
| cdMGS01 | *E. coli* BL21 harboring pETCM01 | | |
| opMGS01 | *E. coli* BL21 harboring pETOM01 | | |
| opMGS02 | *E. coli* BL21 harboring pETOM02 | | |
| opMGS03 | *E. coli* BL21 harboring pETOM03 | | |

^a^ Italic letters indicate homologous sequence for infusion reaction in Gibson cloning. Bold letters indicate restriction sites. Ribosome Binding Site (RBS) is indicated by single-underlined letters. Double-underlined letters indicate a Strep tag or an Asp tag (OM3_For only).

**
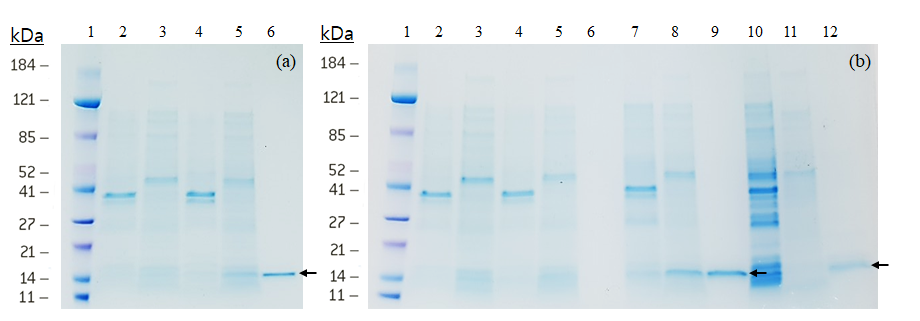
**

**Supplementary Figure S1.** SDS-PAGE for insoluble total protein, soluble total protein and Strep-purified protein expressed from the *E. coli* strains**.** **(a)** Result of cdMGS expression and purification. Lane numbers correspond to: 1, protein marker; 2, MGS00 insoluble; 3, MGS00 soluble; 4, cdMGS01 insoluble; 5, cdMGS01 soluble; 6, cdMGS01 Strep-purified protein. **(b)** Result of opMGS expression and purification. Lane numbers correspond to: 1, protein maker; 2, MGS00 insoluble; 3, MGS00 soluble; 4, opMGS01 insoluble; 5, opMGS01 soluble; 6, opMGS01 Strep-purified protein; 7*,* opMGS02 insoluble; 8, opMGS02 soluble; 9, opMGS02 Strep-purified protein; 10, opMGS03 insoluble; 11, opMGS03 soluble; 12, opMGS03 Strep-purified protein. As the N-terminal Strep tagged opMGS did not express soluble protein, the tag was moved to the C-terminal. Asp tag was also added to the N-terminal for additional solubility of the protein.
